# Editorial: Protozoan parasites in the multi-omics era: present and future

**DOI:** 10.3389/fcimb.2023.1281638

**Published:** 2023-09-14

**Authors:** Francisco Callejas-Hernández, Carlos Robello, José M. Requena

**Affiliations:** ^1^ Center for Genomics and Systems Biology, Department of Biology, New York University, New York, NY, United States; ^2^ Institut Pasteur de Montevideo and Facultad de Medicina, UDELAR, Montevideo, Uruguay; ^3^ Centro de Biología Molecular Severo Ochoa (CSIC-UAM), Departamento de Biología Molecular, Instituto Universitario de Biología Molecular (IUBM), Universidad Autónoma de Madrid, Madrid, Spain

**Keywords:** parasite, omics, disease, NGS, NTD

Parasitic diseases contribute significantly to the global human disease burden. Many of them are classified into the group of Neglected Tropical Diseases (NTDs), largely due to insufficient attention from public funding agencies in developed countries. Collectively, NTDs impact over 1 billion people globally, predominantly in rural areas of low-income countries. At present, there is no vaccine against parasitic diseases of general acceptance (Solana et al., 2022). The currently available chemotherapies, overall, are poor and suffer from serious side effects, limited efficacy, and resistance development.

Therefore, understanding parasites at the molecular level has become essential for designing new strategies to control these devastating diseases. In the last decade, new technological advances have forever changed the way genomes, transcriptomes, proteomes, and metabolomes are analyzed, successfully generating huge amounts of molecular data for parasites, hosts, and their interactions. Furthermore, these technologies (known as ‘omic’ approaches) have become increasingly accessible in terms of cost and time, promoting their implementation in a growing number of specialized research laboratories worldwide (**Figure 1**). Omic studies, including but not limited to genome sequencing, transcriptomics, proteomics, metabolomics, and epigenetics, are contributing to addressing specific biological questions, such as drug resistance, epidemiology, genetic exchange, immune evasion mechanisms, genome organization, and gene expression. Equally important, their application in disease surveillance, disease outcomes, and interactions with hosts and vectors will have positive impact in improving human health.

**Figure 1:**
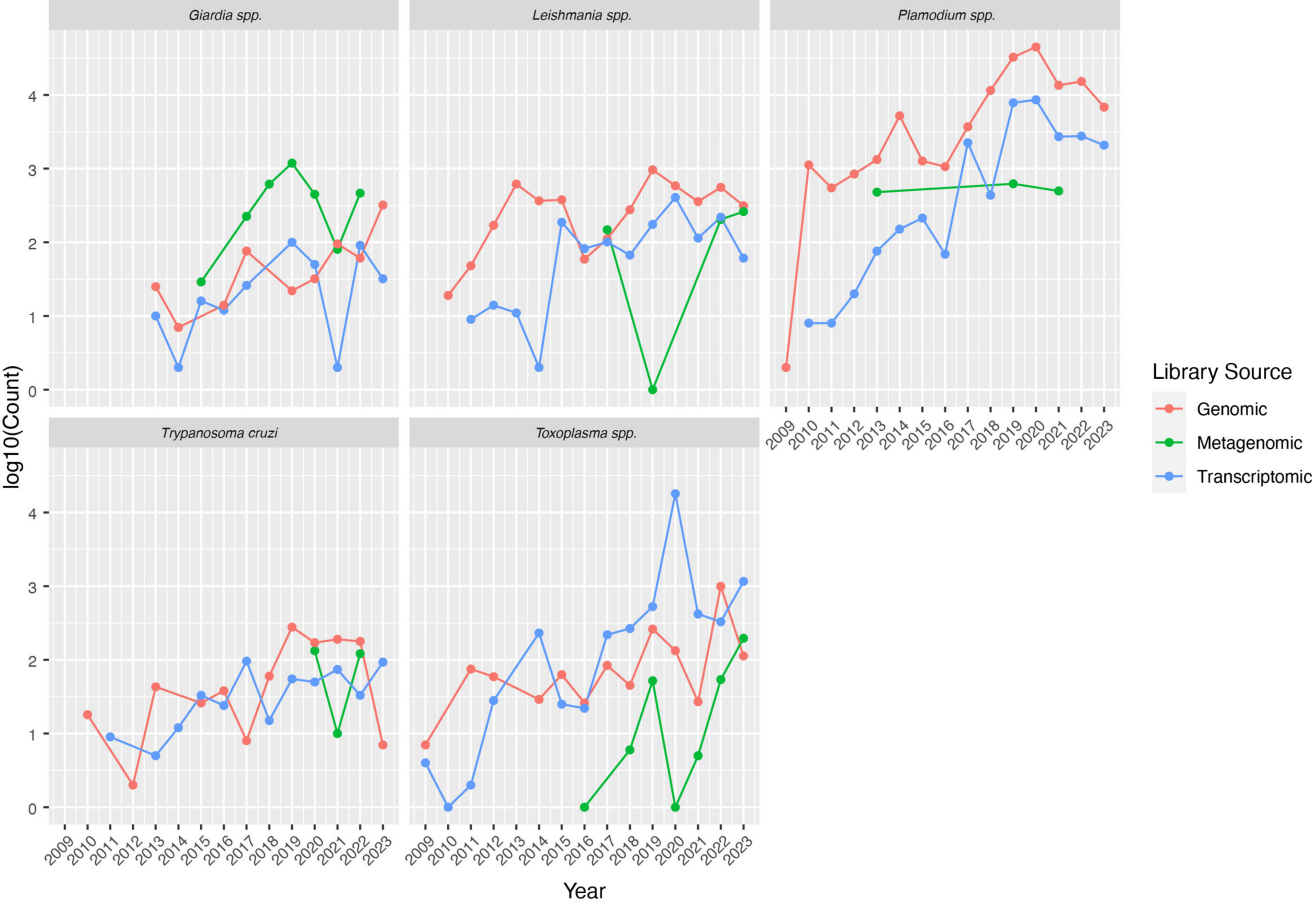
Metadata analysis of the parasites included on this Research Topic and the information available at the sequence read archive (SRA) database on omic studies. The number of SRA entries per year is shown in the Y axis (note that log10 based scale is used for visualization purposes). Metadata were downloaded using the search command from the Entrez Direct tool (NCBI E-utilities).

Thus, this Research Topic was intended to spotlight recent advances in the field of parasite omics applied to study protozoan pathogens. It includes articles focused on parasites with a high impact in human health; four of them deal with causative agents of the so-called NTDs by the World Health Organization (WHO, 2023). These are: *Trypanosoma cruzi*, the causative agent of the Chagas disease, an endemic disease in Latin America but expanded globally through human migrations. Second, *Giardia intestinalis*, the causative of a diarrheal infection named as giardiasis and affecting countries with poor water quality, sanitation, and food safety measures (usually low-income countries). Third, *Toxoplasma gondii*, an apicomplexan parasite causative of toxoplasmosis, a zoonosis of medical and veterinary importance with devastating consequences for those with compromised immunity or during pregnancy [also considered a neglected parasitic infection in the US by the Center for Disease and Control prevention (CDC, 2023)]. Finally, *Plasmodium falciparum*, a second parasite from the apicomplexan phylum, and the causative of the malaria disease, which ranks first among parasitosis.

In the article by Puerta et al., the authors reviewed and discussed mechanisms of antigenic variability used by *T. cruzi* over the time that allows it to chronically live and proliferate inside the host cells and evading the immune response. A special emphasis is laid on the cell-mediated immunity by CD8+ T cells during the chronic phase of Chagas disease, whose presence can be also correlated with cardiomyopathy progress and other disease outcomes. They also recognized the importance of the omics approaches for the discovery of new biomarkers and/or new therapies for disease control. Furthermore, these authors raised the need of assembling reference genomes for the different DTUs (discrete typing units) existing in this parasite, given the remarkable genomic plasticity among them, a factor contributing to varied pathological outcomes (Herreros-Cabello et al., 2020).

Rego et al. conducted a comprehensive comparative analysis of miRNA profiles during the initial stages of *T. cruzi* infection in epithelial cells, cardiomyocytes, and macrophages. Their study, utilizing small-RNA sequencing, unveiled distinct and highly cell type-specific patterns. For instance, three miRNAs (miR-146a, miR-708, and miR-1246) were identified as consistently present in response to *T. cruzi* infection across different cell types, and macrophages were found to produce the strongest response against the infection compared with that elicited by the infection in cardiomyocytes and epithelial cells. Furthermore, in line with expectations for this parasite, the study confirmed the absence of silencing machinery and potential sncRNAs that could mimic host miRNAs.

The article by Rojas et al. is focused on changes in gene expression at seven hours post-infection in intestinal epithelial cells (IECs) with either trophozoites (replicative form) and cysts (transmissive form) of *Giardia intestinalis*. The authors performed dual-RNAseq to identify differentially expressed genes (DEG) potentially involved in the infection process and the establishment of the disease. The results showed a broad repertoire of DEG in the host cells in response to the infection, and some others specific to the parasite life stages. For instance, HES7, HEY1, and JUN (transcription factors), MUC2 and MUC5A (mucins), among others, are preferentially upregulated in the presence of trophozoites, whilst cysts induced a stronger chemokine response (CCL4L2, CCL5 and CXCL5). IECs exhibited an immune response phenotype, marked by DEGs associated with MAPK signaling, apoptosis, cholesterol metabolism, and oxidative stress, among others. While most of the transcriptional changes identified in the parasite correspond to genes of so far unknown function, some others are likely involved in promoting a robust protective phenotype against the oxidative stress generated by the host.

In the apicomplexan parasite *Toxoplasma gondii*, Yang et al. identified three tRNA-specific 2-thiolation enzymes (involved in the s^2^U34 modification of tRNA^Lys^, tRNA^Glu^, and tRNA^Gln^), and analyzed the biological effect of the deletion of one of them (TgMnmA) in the parasite biology and during the infection process. Their results confirmed the importance of this gene during the lytic cycle of the tachyzoite (replicative) stage, more specifically during the apicoplast biogenesis and transcription. Besides, the TgMnmA knockout showed less virulent phenotype, presumably because of intracellular growth defects, and therefore it might represent a good candidate for therapeutic intervention.

Finally, Honfozo et al. constructed a clonal transgenic *Toxoplasma* line expressing the TgSORT-GFP (receptor and transporter involved in the biogenesis of the secretory organelles rhoptries and micronemes) and ROP-mCherry (rhoptry protein localized near the apical end). Subsequently, they used the fluorescent-labeled parasites in a miniaturized image-based phenotype assay to screen over a thousand compounds for their ability to disrupt *T. gondii* polarity, morphology, and intracellular replication. This extensive screening yielded 12 compounds (kinase inhibitors) demonstrating antiparasitic activity, with four of them (Cpd4, Cpd6, Cpd9, Cpd12) being novel discoveries. Interestingly, some of those compounds are also capable of inhibiting *P. falciparium* growth in red blood cells (even at micromolar doses). These findings provide a promising starting point for designing and developing anti-parasitic compounds that exhibit both cross-species and species-specific efficacy.

## Perspectives

Although the multi-omics era is revolutionizing our knowledge on transcriptomes, genomes, proteomes, and more, there still exists a significant disparity in research efforts focused on parasites and the diseases they cause. To illustrate this, we have conducted an analysis of the Sequence Read Archive (SRA) to enumerate the genomics projects dealing with the main protozoan pathogens. Thus, in 2020 the total number of SRA entries for genomic analyses in *Plasmodium* spp. reached a maximum peak of 45 thousand, whereas for *T. cruzi*, it barely reached 400 in 2019 (**Figure 1**). Similar numbers were found for *Giardia spp* and *Toxoplasma spp*, whereas the less studied parasite is *T. cruzi*. To this date, there are 157 genome assemblies available for *Plasmodium spp*, half of them (83) represent complete assemblies (at chromosome-scale), 72 for *Leishmania spp* (43 are complete), 32 for *Giardia spp* (3 are complete), 28 for *Toxoplasma spp* (6 complete, including the type A and B groups) in contrast, no complete assemblies are available for *T. cruzi* (the strains Brazil clone 4, and Y clone 6 released in 2020 (Wang et al., 2021) are nearly complete assemblies, but still contain hundreds of un-scaffolded contigs).

Given the vast genomic variability (sometimes referred as genome plasticity or population dynamics) among parasites, the leading Omic studies are genome-scale analyses, followed by transcriptomics and metagenomics ones (**Figure 1**).

In summary, this Research Topic gathers valuable insights into the contributions of omics technologies to our knowledge of the molecular biology of parasites relevant to human health. An exponential growth in the utilization of such technologies, along with advancements in them (particularly third-generation technologies), is expected to continue in the coming years. While we acknowledge the advances and milestones reached, we remain mindful of the challenges that still lie ahead.

## Author contributions

FC-H: Project administration, Supervision, Writing – original draft, Writing – review & editing. CR: Project administration, Supervision, Writing – review & editing. JR: Project administration, Supervision, Writing – review & editing.
